# Salmagundi

**Published:** 1884-12

**Authors:** 


### SALMAGUNDI.

EDITED BY E. G. BETTY, D.D.S.

DECADENCE OF TEETH.—The decline and fall of American teeth can not but fill the thoughtful patriot with alarm. The average quantity of original teeth owned by an American of the present generation is seven and three-tenths. Comparing this state of things with the teeth of the last generation, and assuming that the same rate of decadence will continue, we find that Americans of the coming generation will have no teeth whatever. It behooves dentists to make hay while the sun shines, for the day when dentists will have no employment except the manufacture and fitting of false teeth is close at hand.

What is the matter with American teeth? It can not be the climate, for the red men who have inhabited this continent from time immemorial have sound teeth, which defy all areas of depression, storm vortices, and other climatic eccentricities. It can not be wholly the form of government under which we live. The Romans and Italians of the Middle Age republics had excellent teeth, and the people of the oldest existing republic in the world, that of San Marino, can not furnish business enough to keep a single dentist from starvation. Whatever objections may be made to republicanism, it is reasonably certain that it has no injurious effect upon the teeth.

When, however, we come to consider the effect of religion upon the teeth, we can easily find facts that serve to show that religion is not without its influence in hastening or retarding the decay of teeth. As a broad, general proposition, it may be safely asserted that Protestant teeth are inferior to Roman Catholic teeth. The finest teeth in the civilized world are the Neapolitan teeth, and the Neapolitian is the most excessive Roman Catholic in existence. The Neapolitan colonists in this city have teeth of the purest white, and of a strength little, if any, inferior to that of steel. Experiments tried with the beefsteak of American commerce and a Neapolitan of Mulberry street, demonstrate that the latter can bite the steak with as much ease as the average American can bite a buckwheat cake. In Spain and Portugal, among the French peasantry and the Roman Catholic Irishmen, sound teeth are the almost universal rule. Of itself, this fact proves nothing, but when taken in comparison with the condition of Protestant teeth it is full of meaning.

The teeth of Protestant Germany, Sweden, and England are far inferior to the teeth of the countries above mentioned. They are, it is true, superior to the American teeth, but they are on the downward road and are doomed to ultimate extinction. In this country, where Protestants are largely in the majority, the melancholy state of the national teeth is notorious, and the conclusion that Protestantism is unfavorable to teeth seems inevitable.

There are, it is true, exceptions to this rule. Calvinism seems to harden the enamel of teeth, and even to favor their growth. Thus the Scotch Calvinists have not only sound teeth, but they seem to have more teeth than they really need. The prevalence of genuine teeth in New England fifty years ago was undoubtedly due to the general belief in Calvinism, and just so far as this belief has disappeared New England teeth have apparently tried to disappear with it. On the other hand, ritualism in the Anglican Church is obviously favorable to teeth, and earnest churchmen can point with pride to the conspicuous merit of the teeth of most of the ritualistic clergy.

Why different religions should have different effects upon the teeth is not verv clear. Possibly the Catholic, by his frequent fasts, rests his teeth and gives them time to recuperate. Possibly the smoke of incense has a preservative effect upon the enamel, or it may be that the absence of political sermons, which are so frequently conducive to grinding of the teeth on the part of dissatisfied hearers, has its share in preserving Catholic teeth from injury. Whatever may be its true explanation, the theory of a connection between religion and teeth is a very interesting one, and certainly deserves to be thoroughly investigated.—*New York Times.*

We give the above to our readers, not because it sheds any light upon the subject of which it treats, but because it is one of those curiosities that Isaac D'Israeli would have treasured up were he still living.

TIRED EYES. People speak about their eyes being fatigued, meaning that the retina, or seeing portion of the brain, is fatigued, but such is not the case, as the retina hardly ever gets tired. The fatigue is in the inner and outer muscles attached to the eyeball and the muscle of accommodation, which surrounds the lens of the eye. When a near object is to be looked at, this muscle relaxes and allows the lens to thicken, increasing its refractive power. The inner and outer muscle to which I referred are used in covering the eye on the object to be looked at, the inner one being especially used when a near object is to be looked at. It is in the three muscles mentioned that the fatigue is felt, and relief is secured temporarily by closing the eyes or gazing at far distant objects.

The usual indication of strain is a redness of the rim of the eyelid, betokening a congested state of the inner surface, accompanied with some pain. Rest is not the proper remedy for a fatigued eye, but the use of glasses of sufficient power to render unnecesary so much effort to accommodate the eye to vision.—*Exchange.*

Dentists will do well to think about this, as very many often suffer severely from fatigue of the eyes after protracted operations at the chair.

---

THE Ohio State Dental Society, before it disbanded, passed a resolution turning over to the *Ohio State Journal* and the REGISTER all its papers, essays, etc., including its stenographic report. Both of the above named journals had reporters on hand, intending to publish their reports in the December number of each journal. It is intended now, however, to wait till January, as the reports can can be made fuller by combining with the short-hand report. This action is all the more desirable for the reason that the Nineteenth Annual Meeting of the O. S. D. S. will have no published transactions other than the reports to be so published.

All members of the society, wishing to complete the files of the transactions, will do well to order the January number of the REGISTER for 1885. We would be glad to have them order it for the entire year while they are about it, for, "now's the time to subscribe."

"It's an ill wind that blows nobody good."

---

According to the *Popular Science News*, to obviate hereditary tendency to disease in the young, "wash them, air them, and *iron* them."

---

Dr. Leidy, of Philadelphia, has discovered worms in ice. This may be accounted for, perhaps, by the unpleasant fact that too many cocktails will give a fellow the snakes."

---

A French woman is making money by pulling teeth, at Newark, N. J. Men flock to her to have their ivories pried out, as they did to female barbers when they were a novelty.—*Daily Paper*.

---

It may seem a little "too previous," but we wish one and all a Merry Christmas, and we might add a Happy New Year; for, the Register will not meet its good friends again until the holidays shall have come and gone.

---

A Philadelphia dentist publicly favored the admission of women to the ranks of his profession. The next thing we heard was that a woman in Michigan extracted her husband's front teeth with a base ball bat.—*Daily Paper*.

---

We earnestly hope that the reorganized State Society will secure a registry law and that hereafter, graduation from a dental college in good repute will be the standard required of all assuming to practice dentistry in Ohio. That's the way to elevate the dignity of the profession.

---

"Recent statistics on the comparative longevity of the sexes show that under fifteen years there are more boys than girls, but over seventy-five years there are more women than men; and from the ages of ninety to one hundred the proportion is about three to two in favor of the women."

Dr. Watt was present at Columbus, feeling so well that he took an active part in the reorganization of the State Society, his name heading the list of incorporators. We hope to see him at Chillicothe next October, armed with "the best effort of his life," in the way of one of his inimitable essays.

---

As will be seen by reference to the proceedings of the "Ohio State Dental Society, as Re-organized," published elsewhere in this issue of the Register, the Phœnix has arisen from her ashes with new vigor, hopes, and ambitions. We wish her success, and will lend every effort to enable her to accomplish it.

---

How to combine business and pleasure was shown by a placard in Chicago, on Tuesday, Nov. 4th.

VOTE FIRST!!
Then Come Up Stairs and Get Your
Teeth Pulled.

---

Migratory neuralgic pains can be demolished with Iodide of Potash in the following proportion:

R. Potassi Iodide ʒii.

Aq. Dest ℥ss.

M. ft. Sol.

Sig. Take four drops in a wine-glass of water three times per day.

---

The following ancient pun is "going" the rounds again, for the benefit of the rising generation we presume:

Going & Gonne was the style of a well-known banking-house in Ireland, and on their failure in business some one wrote:

"Going & Gonne are now both one,
For Gone is Going, and Going's gone."

---

Dr. N. S. Hoff, of Cincinnati, and Miss Addie L. Chickering, of West Boylston, Mass., were married at the latter place on the 23rd of October last. The bride is a daughter of the late Prof.

J. B. Chickering, founder of "Chickering's Institute," of Cincinnati; an institution for preparing candidates to enter the great universities. Dr. Hoff and his wife enjoy the hearty congratulations and best wishes of many friends.

---

A WRITER in the *Canada Medical Record* says he has greatly relieved attacks of epilepsy by treating with nitro-glycerine, but has as yet effected no entire cure. It has been our impression until this time that nitro-glycerine usually accomplished a permanent cure of every complaint in the person to whom it was applied. Possibly the good doctor does not administer a heavy enough charge—reserving that for the bill. J. C.

---

PROSTHETIC DENTISTRY does not seem to be as badly on the wane as many suppose; instance the many improved continuous gum furnaces that have lately been brought to the notice of the profession. It is a significant fact that there has been such a great reduction in the size of furnaces. thereby doing away with the cumbrous masonry of by-gone years, together with the heat and long-continued firing up that was so dreaded. Coal gas now bids fair to supercede coal, and the time is coming when it will almost be possible for the dentist to work at his chair while a piece of continuous gum is baking in the gas furnace alongside the operating case.

---

To fathers and mothers surrounded by luxury and flattered with the precocity of their infants, which they are stimulating to the last degree, we say: Do not under peril encourage this brilliancy, which is now so charming; let the mind stagnate rather. For the first seven years of life give concern *only* to his morals and to his physique. Nourish him as you would nourish an animal from which you desired the finest development, stimulating *only* his moral nature, and his intellect will take care of itself. Thus, if he have no hereditary taint, will be laid the foundation of a splendid specimen of his race.—*Norman W. Kingsley.*

